# Accumulation of Potentially Toxic Elements and Bioindicator Potential of Necrophagous Flies in Exposed Municipal Wastes

**DOI:** 10.1007/s00244-026-01186-5

**Published:** 2026-03-23

**Authors:** Kittikhun Moophayak, Chutarat Saengkul, Puntaree Taeprayoon, John Pichtel, Siwaporn Premmanee, Chonthicha Thumjan, Chalida Thala, Piyathap Avakul, Weeradej Meeinkuirt

**Affiliations:** 1 Medical and Agricultural Fly Research Unit, Mahidol University, Nakhonsawan Campus, Nakhonsawan, 60130 Thailand; 2 Occupational Health and Environmental Research Unit, Mahidol University, Nakhonsawan Campus, Nakhonsawan, 60130 Thailand; 3 Agricultural and Environmental Utilization Research Unit, Mahidol University, Nakhonsawan Campus, Nakhonsawan, 60130 Thailand; 4 Geology, and Natural Resources, Ball State University, Environment, Muncie, IN 47306 USA; 5 Water and Soil Environmental Research Unit, Mahidol University, Nakhonsawan Campus, Nakhonsawan, 60130 Thailand; 6 Department of Biology, Faculty of Science, Mahasarakham University, Kantharawichai, Maha Sarakham 44150 Thailand; 7 Academic and Curriculum Division, Mahidol University, Nakhonsawan Campus, Nakhonsawan, 60130 Thailand

## Abstract

To assess fly diversity, abundance, and accumulation of potentially toxic elements (PTEs) in tissue, necrophagous fly communities were collected from open waste bins and landfills in western Thailand. Highest species richness was recorded in Mae Pa and Panlan, each with 11 species; however, Mae Pa exhibited a higher Margalef’s richness index and significantly greater abundance (4,874 individuals) compared to Panlan (314 individuals). Khaothong showed lowest richness with 5 species and a moderate abundance of 944 individuals. Several fly species accumulated considerable PTEs from municipal wastes. Cadmium and zinc were among the most enriched of the PTEs, showing high *CF* values in the study locations ࣧ 15.25 at Ban Makluea, 15.82 at Pha De and 30.43 at Khaothong, respectively. Principal component analysis indicated that *Atherigona* spp. and *Musca sorbens* were highly correlated with cadmium, aluminum, iron, lead and chromium, particularly in Pha De and Mae Pa, respectively. *Musca sorbens* accumulated the highest concentrations of Cd (278 mg kg^−1^) and Zn (101 g kg^−1^) in Pha De. This study suggests that *Musca* species of necrophagous flies, despite the need of further research for confirmation, are effective as potential bioindicators of Cd and Zn, especially in polluted environments.

**Supplementary Information:**

The online version contains supplementary material available at 10.1007/s00244-026-01186-5.

## Introduction

Worldwide generation of municipal solid waste (MSW) has increased in recent years to the rapid increase of the global population together with improvements in consumer lifestyles (Bottausci et al. 2022; Chen et al. 2020). MSW occurs in various forms including general rubbish (e.g., rubber, textiles, plastic bags), recyclable materials (metals, glass, paper, cardboard), and degradable waste (food scraps, garden and lawn clippings). These wastes, when improperly managed, polluted soil, groundwater and surface water via release of inorganic and organic components (e.g., metals, salts, nutrients, hydrocarbon fuels) (Dagwar and Dutta 2024). Potentially toxic elements (PTEs) are among the most observed pollutants released in urban, suburban and rural areas, thus indicating a need for improved management strategies for prevention of PTE exposure (Sellamuthu et al. 2025). Bioindicators occur in numerous forms, such as plants (e.g., mangrove and macrophytes), animals (e.g., insect, birds and fish) and microorganisms (e.g., bacteria and fungi); these indicators play crucial roles in monitoring toxic elements in air, water and soil (Manickavasagam et al. 2019; Stankovic et al. 2014). Insects have been reported as effective indicators for identifying and tracking environmental contaminants; thus encouraging interest and effective measures for pollutant mitigation thus encouraging interest and effective measures for pollutant mitigation (Bandhaw et al. 2025). A recent review evaluated the multidisciplinary roles of bees (Hymenoptera, Anthophila) during pollination, including their adhesion efficiency to PTEs, plastics and various organic and inorganic chemical compounds. Bees were subsequently harnessed as bioindicators for particulate matter (PM) (Meacci et al. 2025).

Waste management practices are effective in developed countries, as the infrastructure is well-established and often employs advanced technologies for waste processing, separation, and ultimate disposal. However, developing countries, including Thailand, do not as yet incorporate such practices; for example, sewage solids and MSW are typically disposed in the same location. This practice is partly due to lack of available land and the high costs associated with an effective waste management infrastructure. Furthermore, certain municipal waste landfills have not been properly designed (for example, having inadequate or no liners), and some are not properly controlled, resulting in leachate seepage to groundwater and surface water (Sewak et al. 2021). Landfill leachate is often hazardous due to substantial concentrations of such PTEs as chromium (Cr), mercury (Hg), and cadmium (Cd) (Talalaj 2015). Such PTEs pose considerable risk to health of humans and other biota because leachate may enter irrigation water and soil, leading to uptake and accumulation by crop plants (Agbemafle et al. 2020).

Many insects (Class Insecta), despite having diverse bioaccumulation capacities during different life cycles, body integument structures and behavioral patterns, are excellent bioindicators of environmental contamination for both aquatic and terrestrial ecosystems (Ramola et al. 2024). Several insects such as dragonflies (Order Odonata), butterflies (Order Lepidoptera) and grasshoppers (Order Orthoptera) may be used as bioindicators of PTEs as they are documented to tolerate and proliferate in habitats contaminated with PTEs and other pollutants (Azam et al. 2015). Insect species of Order Diptera, representing most flies, have been examined for bioaccumulation capacities of toxics (e.g., heavy metals), providing valuable data associated with human and ecosystem health (dos Santos Junior et al. 2023). This implies that flies possess effective traits for adaptation to highly contaminated locations (Diener et al. 2015b). Necrophagous flies are among the most effective bioindicator species of Diptera for qualitative assessment of environments contaminated by PTEs (Moophayak et al. 2024). Although flies are essential for the decomposition of organic materials and nutrient cycling in the environment, they are also beneficial for assessing anthropogenic impacts on ecosystems; differences in such capabilities are evident via investigation of species composition, diversity and abundance (Jahan et al. 2025).

Necrophagous flies are proposed as bioindicators for polluted sites as they thrive in landfills and open dump sites (Hore and Banerjee 2017). Many species of necrophagous flies in the Dipteran families (e.g., Calliphoridae, Muscidae and Sarcophagidae), including *Chrysomya albiceps* (Wiedemann), *Cochliomyia macellaria* (Fabricius), *Chrysomya megacephala* (Fabricius), and *Chrysomya putoria* (Wiedemann), have experienced a decline in abundance in human-disturbed areas in the Ibera wetlands of Argentina. In contrast, *Oxysarcodexia thornax* (Walker) is common, making it a suitable indicator in urban ecosystems since it is highly adapted to anthropogenic activities (Dufek et al. 2016).

Necrophagous flies that survive in environments contaminated with PTEs are valuable bioindicators, as their presence, abundance, and physiological condition reflect local environmental health (Moophayak et al. 2024). By accumulating PTEs in their tissues, these flies provide a practical means for monitoring pollution level and tracking the extent of environmental contamination over time. Their role is particularly important in ecological assessments, offering insights into the distribution and bioavailability of PTEs in degraded ecosystems. Furthermore, their ability to persist in polluted habitats highlights mechanisms of ecological resilience and adaptation to chemical stressors, thus contributing to a deeper understanding of ecosystem response to anthropogenic disturbances (Mendes et al. 2021).

In the reported study, necrophagous fly communities were captured near open waste containers and landfills in Tak and Nakhon Sawan Provinces, Thailand. Several diversity indices were computed to estimate the abundance of each fly species based on location. The source(s) of PTEs in the selected environment and in biota ultimately reflect the contribution of anthropogenic activities. Such data can elucidate PTE spatial distribution of the study location and be used to evaluate ecological risk based on PTE level (Li et al. 2022). Due to the paucity of reports on this topic, the current study was conducted as a preliminary investigation to observe the abundance of diverse necrophagous fly species, including diversity and bioindication potential with respect to accumulated PTEs based on location in two provinces in Thailand. Substantial PTE accumulation in fly tissue may provide insights into how PTE contamination affects fly community species richness and abundance in harsh environments. The current study serves as a baseline report for PTE contamination in fly tissue collected from exposed municipal wastes in Thailand. Ecological risks were also assessed via contamination potential (pollution index) of PTEs, identifying bioindication capacity of certain necrophagous flies.

## Materials and Methods

### Study Site

**Fig. 1:**
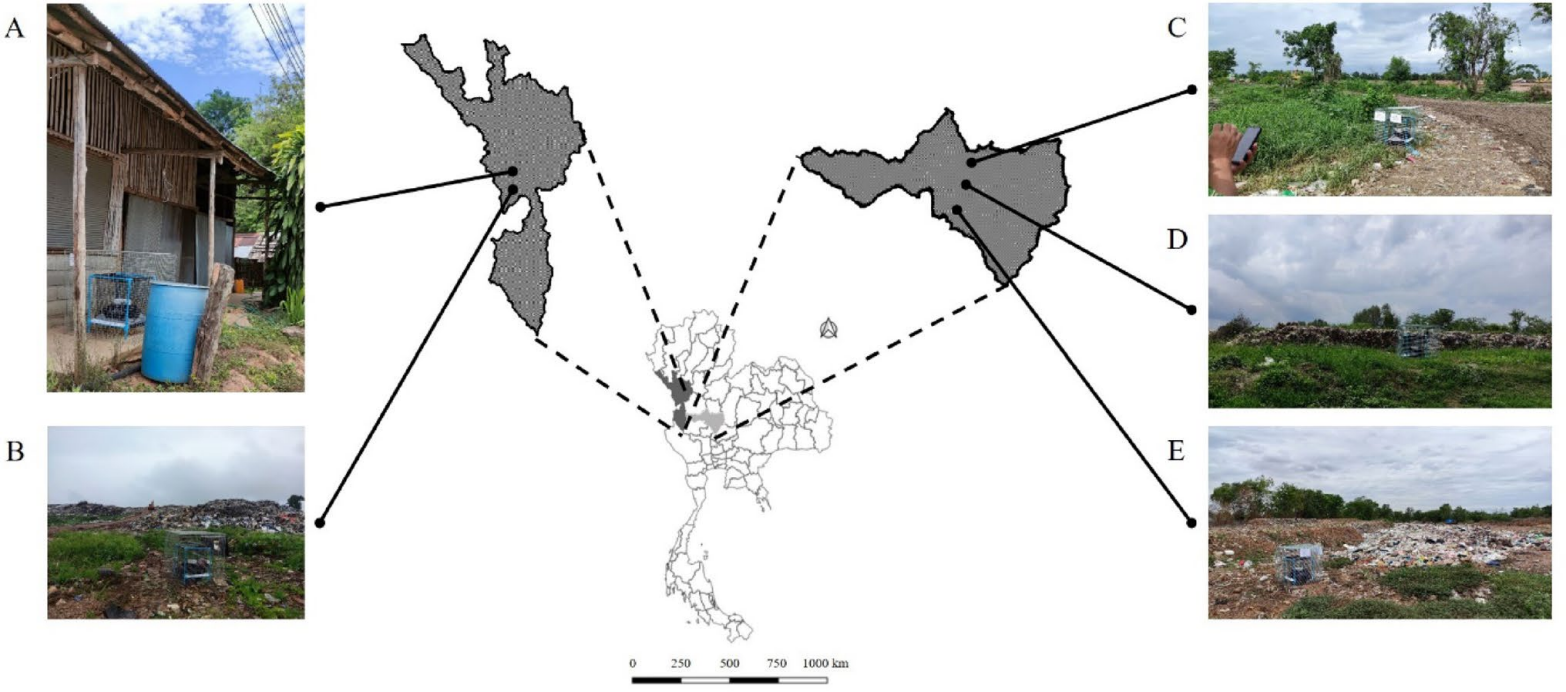
Map of Thailand indicating five study locations at Tak and Nakhon Sawan Provinces: open waste bins at Pha De subdistrict, Mae Sot District, Tak **A**; landfill at Mae Pa subdistrict, Mae Sot District, Tak **B**; landfill at Ban Makluea subdistrict, Muang District, Nakhon Sawan **C**; landfill at Panlan subdistrict, Chumsaeng District, Nakhon Sawan **D**; landfill at Khaothong subdistrict, Phayuha Kiri district, Nakhon Sawan **E**

Necrophagous fly communities were sampled from five locations comprising one open waste bin and four landfills. The open waste bin was located at Pha De Subdistrict, Mae Sot District, Tak Province. Landfill sites were located at Mae Pa Subdistrict (Mae Sot District, Tak Province), Ban Makluea Subdistrict (Muang District, Nakhon Sawan Province), Panlan Subdistrict (Chum Saeng District, Nakhon Sawan Province), and Khaothong Subdistrict (Phayuha Khiri District, Nakhon Sawan Province) (Fig. 1). These sampling sites were designated as Pha De, Mae Pa, Ban Makluea, Panlan, and Khaothong, respectively (Table S1). The collection sites were situated at 256, 200, 28, 27, and 70 m above mean sea level, respectively. The Pha De subdistrict is a small village in the Mae Tao River basin, where local streams and soil are heavily contaminated with Zn and Cd (Taeprayoon et al. 2023).

Mean temperature, mean rainfall, and total rainfall during the dry season (March to May) were 29.4 °C, 200.8 mm, and 864.1 mm for Tak Province, and 30.1 °C, 108.4 mm, and 771.5 mm for Nakhon Sawan Province. All meteorological data were obtained from the Tak and Nakhon Sawan Meteorological Stations. The study was conducted during the peak active period of flies in the dry season, late April to early May 2022.

## Fly Collection and Identification

Necrophagous flies were captured using a bait-trap technique. A collection trap (50 cm ⋅ 50 cm ⋅ 50 cm) equipped with a removable net (430 holes/cm^2^) was set for 3-h periods in the morning (9–12 am) and afternoon (1–4 pm), in triplicate at each site. According to Moophayak et al. (2017), fly activity peaks between 9:00 AM and 12:00 PM, while egg-laying and colonization were most frequent from 1:00 PM to 4:00 PM, highlighting these periods as critical for observing. A box containing 300 g of 2–3 day-old spoiled pork was placed under the trap and used as bait. The measured concentrations for the spoiled pork were Cd (0.028 mg kg^−1^), Pb (0.004 mg kg^−1^), Cu (12.3 mg kg^−1^), Zn (35.3 mg kg^−1^), Fe (97.3 mg kg^−1^), Al (0.35 mg kg^−1^), Cr (0.04 mg kg^−1^), Ni (0.013 mg kg^−1^), and Mn (3 mg kg^−1^). In addition, a five-sided metal shelter measuring 1.20 m ⋅ 1.20 m ⋅ 1.20 m with a mesh size of 2.5 cm ⋅ 2.5 cm was placed over the collection trap to protect the bait from large scavengers (rats, birds, dogs) (Fig. 1A-E). Trap dimensions and the use of spoiled pork meat as bait were selected based on previous studies that demonstrated their effectiveness in attracting a wide range of necrophagous fly species under natural conditions. The chosen trap size ensured adequate space for fly entry and retention, while spoiled meat was used due to its proven efficacy in mimicking natural carrion and consistently attracting muscid, calliphorid, and sarcophagid flies (Moophayak et al. 2017).

At each collection event, the net containing captured flies was removed from the trap and placed into an ethyl acetate box for 20 min to anesthetize all flies. Specimens were placed separately in plastic collection bags and stored at 4 °C until identification. Fly specimens were sorted under a stereo light microscope into families of necrophagous types according to morphological characters using the taxonomic key of Triplehorn and Johnson (2005). Necrophagous Calliphoridae were subsequently identified to the species level using the key of Kurahashi and Bunchu (2011). Flies of Muscidae were identified according to the keys of Moophayak et al. (2011) and Tumrasvin and Shinonaga (1977, 1978, 1982), and male flies of Sarcophagidae were identified according to the key of Kurahashi and Samerjai (2018). Two to five identified fly specimens were pinned as reference while the remaining specimens were stored in paper bags until analysis to determine PTE concentrations. The study protocols have been reviewed and approved by the Ethics Committee of the National Animal Center (Salaya Campus, Mahidol University, Bangkok, Thailand) for research on animal subjects (COA No. MU-IACUC 2022/010).

In this study, the Jaccard index (*Cj*) was used to compare the similarity of necrophagous fly species in the study locations by using the following formula:

$$Cj=\frac{a}{a+b+c}$$

where *a* is the number of species found in all study locations, *b* or *c* is the number of species found in one specific area. The similarity coefficients indicate the co-occurrence of necrophagous fly species in all study locations (Whiteson 2009).

## Soil Collection

Soil samples were collected from the surface 20 cm using a stainless-steel soil sampler probe at the locations where flies were captured, from the four corners and the center of the sampling area, and thoroughly homogenized to obtain a representative composite sample. A composite soil was placed in a paper bag and delivered to the laboratory for the determination of PTE concentrations.

## Potentially Toxic Element Analysis in Flies and Soil

Fly samples were thoroughly washed, first with deionized water, then with acetone, and finally with 1% HNO_3_. Sonication was used for 5 min in each solution. The samples were then sonicated again and rinsed twice with deionized water (DI water). Fly specimens and soil material were then oven-dried at 60 °C for 2–5 d, and the dry weight was recorded. The dried specimens were ground with an agate mortar and pestle and sieved through a 2-mm mesh sieve. A 0.5 g (dry weight) sample of fly specimens—or at least five individuals per species—was transferred to a Teflon digestion vessel for acid digestion. The digestion mixture consisted of 1 mL of 30% hydrogen peroxide (H_2_O_2_) and 7 mL of 70% nitric acid (HNO_3_; TraceMetal grade). Digestion was carried out using a microwave digestion system (ETHOS One; Milestone Inc.) with a two-step program: a first step at 200 °C for 10 min, followed by a second at 200 °C for 15 min. Each sample was prepared in triplicate. Soil samples were air-dried, ground to pass a 2-mm mesh sieve, and thoroughly mixed. A 0.5 g portion of each sample was transferred to a digestion vessel. The soil was treated with 3 mL of concentrated 70% HNO₃ and 9 mL of 37% hydrochloric acid (HCl; TraceMetal grade) and subjected to microwave digestion using the same system. The microwave program consisted of two steps: 200 °C for 10 min in the first step, and 200 °C for 20 min in the second. Digested fly specimens and soil samples were filtered using Whatman no. 42 ashless filter paper and then adjusted to a final volume of 25 mL using Milli-Q water. Concentrations of PTEs (Al, Cd, Cr, Cu, Fe, Mn, Ni, Pb, and Zn) in each digested sample were determined using a graphite furnace atomic absorption spectrophotometer (GFAAS; AAnalyst 200, Perkin Elmer, USA).

Each analytical batch for quantitative analysis consisted of seven calibration standards, blanks, and samples. Procedural blanks and laboratory controls were processed identically to the samples. Blanks were measured both before and after every set of 20 samples to monitor potential contamination and ensure data accuracy. Also, procedural blanks spiked with the elements at three different concentrations within the covered linear dynamic range were used to assess analytical accuracy. NIST SRM 2710a Montana soil was chosen as the soil standard reference material for the development of acceptable analytical methods and for determining the reliability of results. The percentage recovery of samples ranged from 93.4 to 109.5%.

The instrument was calibrated using single-element standard solutions (Merck) over a concentration range of 0.1–3 mg L^−1^. A blank (1% HNO_3_) was included, and calibration curves were constructed using least-squares regression, with a mid-range standard analyzed every 10–20 samples to verify instrument stability. The limits of detection (LOD) and limits of quantification (LOQ) for the analyzed metals were as follows: Cd (0.005 and 0.015 mg L^−1^), Zn (0.010 and 0.030 mg L^−1^), Ni (0.008 and 0.024 mg L^−1^), Cr (0.006 and 0.018 mg L^−1^), Pb (0.007 and 0.021 mg L^−1^), Al (0.020 and 0.060 mg L^−1^), Fe (0.020 and 0.060 mg L^−1^), Mn (0.005 and 0.015 mg L^−1^), and Cu (0.010 and 0.030 mg L^−1^), respectively. Typical instrumental parameters for the AAnalyst 200 (PerkinElmer) were set according to the manufacturer’s guidelines, including a hollow cathode lamp for each element, a slit width of 0.7 nm, an air–acetylene flame, and optimized burner height and gas flow rate for maximum sensitivity and stability. The wavelength (nm) and lamp current (mA) used for each metal are provided in Table S2.

All glassware and plasticware used for sample preparation and analysis were initially washed with laboratory detergent (Extran MA 02 Liquid, Merck), followed by overnight soaking in the same detergent solution in an acid-cleaned plastic container. They were then rinsed several times with double-distilled water, followed by acid-washing in 10% HNO₃ (Merck) overnight. Finally, all materials were thoroughly rinsed with ultrapure Milli-Q water to minimize potential metal contamination.

## Potentially Toxic Element Pollution Indices

Geoaccumulation index (*I*_*geo*_).

The *I*_*geo*_ was originally defined by Müller (1969) and applied for analysis of bottom sediments; however, this index can also be used to assess the intensity of PTE contamination in terrestrial, aquatic, and marine ecosystems (Gupta et al. 2014). In the current study, the *I*_*geo*_ was used to quantify the degree of soil pollution. The index is expressed as follows:

$${I}_{geo}={\mathrm{L}\mathrm{o}\mathrm{g}}_{2}\left[\frac{{C}_{n}}{{1.5B}_{n}}\right]$$

where *C*_*n*_ is the measured concentration of the PTE in soil (*n*), *B*_*n*_ is the geochemical background concentration of the PTE (*n*), and a constant factor 1.5 is the background matrix correction factor that can lessen the impact of variations in background values caused by lithogenic influences in soil. According to Müller (1981), *I*_*geo*_ values are classified as follows: Class 0 (*I*_*geo*_ ≤ 0) indicates uncontaminated; Class 1 (0 < *I*_*geo*_ ≤ 1) uncontaminated to moderately contaminated; Class 2 (1 ≤ *I*_*geo*_ ≤ 2) moderately contaminated; Class 3 (2 ≤ *I*_*geo*_ ≤ 3) moderately to heavily contaminated; Class 4 (3 ≤ *I*_*geo*_ ≤ 4) heavily contaminated; Class 5 (4 ≤ *I*_*geo*_ ≤ 5) heavily to extremely contaminated; and Class 6 (≥ 5) extremely contaminated.

The background concentrations (*B*_*n*_) used in the *I*_*geo*_ calculation were derived in part from data reported by Taeprayoon et al. (2023), as these PTE concentrations are representative of the study locations, which were assessed prior to the present study. The low concentrations at these locations suggest minimal influence from anthropogenic or mining activities.

Enrichment factor of the PTEs in soil (*EF*).

The *EF* is a tool for determining level of PTE pollution in soil from natural and anthropogenic sources. The *EF* was calculated using iron (Fe) as the normalizing element for estimating *EF* values. The index was calculated using the equation:

$$EF=\frac{{\left(\frac{PTE}{Fe}\right)}_{Sample}}{{\left(\frac{PTE}{Fe}\right)}_{Background}}$$

Adaikpoh (2013) described *EF* values to be interpreted as: *EF* ≤ 2 indicates deficiency to minimal enrichment; 2 ≤ *EF* ≤ 5 is moderate enrichment; 5 ≤ *EF* ≤ 20 is significant enrichment; 20 ≤ *EF* ≤ 40 is very high enrichment; and ≥ 40 is extremely high enrichment.

Contamination factor (*CF*).

Contamination factor (*CF*) is the ratio of the particular PTE concentration in soil to its measured background value. The *CF* is defined by:

$$CF=\frac{{C}_{n}}{{C}_{bn}}$$

Where *C*_*n*_ is the PTE concentration in soil (*n*) and C_*Bn*_ is the background value for the studied PTE (*n*). The *CF* is classified into four groups: *CF* ≤ 1 denotes low contamination; 1 ≤ *CF* ≤ 3 is moderate contamination; 3 ≤ *CF* ≤ 6 is considerable contamination; and ≥ 6 is extremely high contamination (Hakanson 1980).

Pollution load index (*PLI*).

The *PLI* is used to calculate the degree of PTE pollution level in soil and was calculated by:

$$PLI= \sqrt[n]{{CF}_{PTE1}+{CF}_{PTE2}{+{CF}_{PTE3}+...+CF}_{PTEn}}$$

where *n* is the number of PTEs studied. A value of *PLI* ≥ 6 indicates polluted and *PLI* ≤ 1 is unpolluted (Kükrer et al. 2014).

### Diversity Indices

Shannon-Wiener diversity index (*H′* ).

The *H′* is used to assess level of diversity of fly species in a community (Shannon 1948). The Shannon-Wiener diversity index was calculated using:

$${H}^{{\prime}}=-\sum\left(\frac{Ni}{N}\right)\mathrm{l}\mathrm{n}\left(\frac{Ni}{N}\right)$$

where *N* is total number of individual organisms in the sample across all species and *Ni* is the number of each species. *H′* values of ≤ 1, 1–3, and ≥ 3 indicate low, moderate and high diversity, respectively.

Simpson abundance index (*D′* ).

The *D′* is a weighted arithmetic mean of proportional abundances (Zhu et al. 2021). It was calculated according to:

$${D}^{{\prime}}=1-\sum{\left(\frac{Ni}{N}\right)}^{2}$$

where *Ni* is the abundance of the *i*th species in a location and *N* is the total number of all species living in the same location.

Pielou’s evenness index (*J*).

The *J* measures the evenness of each fly species within a community, calculated based on number of individuals (Zhu et al. 2021). The index was calculated from:

$$J={H}^{{\prime}}/\mathrm{l}\mathrm{n}S$$

where *S* is total number of species. The evenness index value is categorized as: 0 < *J* < 0.5, depressed community, 0.5 < *J* < 0.75, unstable community, and 0.75 < *J* < 1, stable community (Krebs 1989).

Margarlef’s richness index (*D*).

The *D* is used to measure the species richness of organisms, while the degree of the index reflects level of pollution (Margalef 1958). The index was calculated according to the equation:

$$D=\frac{(S-1)}{\mathrm{l}\mathrm{n}N}$$

*D* values of < 1, 1–3 and > 3 indicate low, moderate and high species richness, respectively.

Dominance index (*Y*).

Dominant fly species were determined by calculating the *Y* within a community or habitat:

$$Y=\sum{\left(\frac{Ni}{N}\right)}^{2}$$

where *Ni* is the number of individuals in the *i*^th^ species, and *N* is the total number of individuals of all fly species collected during the study period. Dominance index values are categorized as: 0 < *Y* < 0.5, low dominance, 0.5 < *Y* < 0.75, moderate dominance, and 0.75 < *Y* < 1, high dominance (Ulfah et al. 2019).

### Data Analysis

All statistical data were analyzed using R ver. 2.15.1 (RStudio-Team 2015). One-way analysis of variance (ANOVA) was performed to determine variations in such data as *PLI* index, PTE concentration in fly species and soil properties in each location. The least significant difference (LSD) *post hoc* test was set at the 95% confidence level (*p* < 0.05). To compare means of PTE accumulation in soil samples and fly tissue, a boxplot was constructed in R using package ‘ggplot2’ to represent *p*-values from the Kruskal-Wallis non-parametric test. An alluvial plot was created to illustrate categorical fly and PTE variables using R-package ‘ggalluvial.’ Principal Component Analysis (PCA) was performed in R using the ‘FactoMineR’ and ‘factoextra’ packages to visualize the abundance patterns of necrophagous fly species that accumulated PTEs, in relation to the corresponding PTE concentrations in soil across different locations.

## Result

### Potentially Toxic Element Concentrations and PTE Pollution Index in Soils

**Fig. 2:**
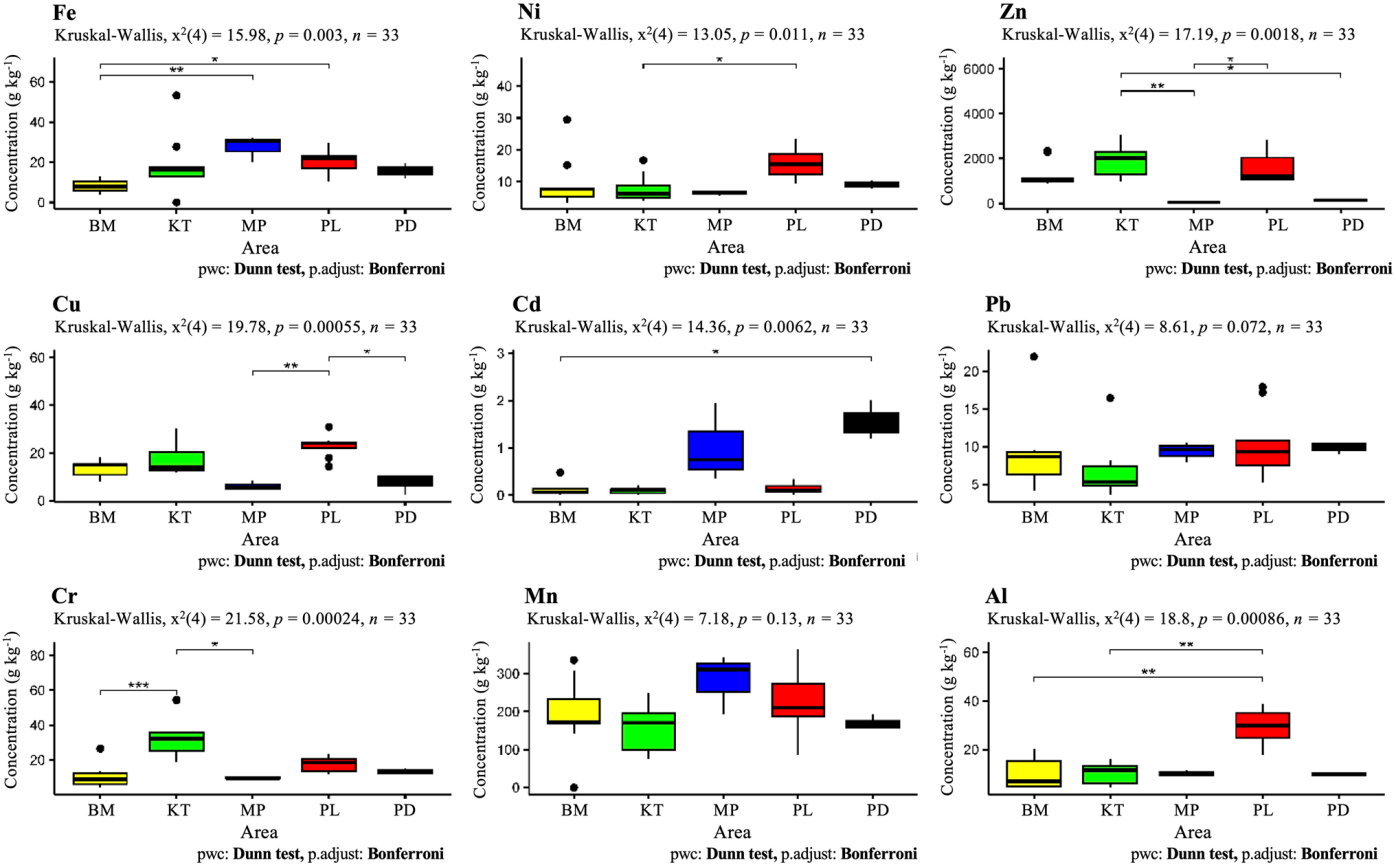
Boxplot from five sampling locations showing concentrations of each PTE in soil. *p* * < 0.05; *p* ** < 0.01; *p* *** < 0.001; dots represent outliers. BM: Ban Makluea, KT: Khaothong, MP: Mae Pa, PL: Panlan and PD = Pha De

Concentrations of PTEs (Al, Cd, Cr, Cu, Fe, Mn, Ni, Pb and Zn) varied with subdistrict in the two provinces (Fig. 2, Table S3). Iron and Al had highest mean concentrations, ranging from 8.3 to 28 g kg^−1^ and 9.9 to 29 g kg^−1^ while Cd (0.1 to 1.6 g kg^−1^) and Pb (6.8 to 10 g kg^−1^) had lowest mean concentrations, respectively. PTE concentrations varied with location. The iron concentration was significantly (*p* < 0.05) highest (28 g kg^−1^) in Mae Pa (Tak) while Ban Makluea (Nakhonsawan, NS) had lowest Fe concentration (8.3). Iron concentrations in both subdistricts (20 g kg^−1^ at Panlan and 23 g kg^−1^ at Khaothong) of NS province were also high but relatively lower than those of Mae Pa. In the case of Al, the Panlan subdistrict had the highest concentration (29 g kg^−1^) (*p* < 0.05) while Pha De had lowest concentration (9.7 g kg^−1^). Overall, Mae Pa subdistrict of Tak province had the significantly highest concentration of Fe (28 g kg^−1^) and Mn (282 mg kg^−1^) while the concentration of Al (29 g kg^−1^), Cu (23 mg kg^−1^), Ni (16 mg kg^−1^), and Pb (10 mg kg^−1^) were relatively or significantly highest in the Panlan subdistrict of Nakhonsawan. In addition, Cd concentration (1.6 mg kg^−1^) was highest in Pha De subdistrict of Tak whereas Khaothong in Nakhonsawan province had significantly highest concentration of Cr (33 mg kg^−1^) and Zn (1,832 mg kg^−1^). Ban Makluea was the only subdistrict in this study having no significantly highest concentration of PTEs, indicating that contamination was relatively low compared to the other locations.

The pollution indices revealed differing index values of PTEs based on location (Table 1). According to the *I*_*geo*_ index, Zn had the most expressed intensity, especially in all sampling locations in Nakhonsawan province; *I*_*geo*_ values ranged from 3.31 to 4.3, which indicate some degree of Zn pollution. Both *CF* and *EF* values of Zn in these locations were proportionately designated as extremely high enrichment; for example, *CF* values ranged from 14.9 to 30.4 while *EF* values ranged from 25.6 to 64 in those areas. Cadmium had the second most elevated pollution index values in all tested locations, after Zn. The pollution indices in locations in Tak province were high (2.4 to 3.4 *I*_*geo*_, 13.18 to 35.4 *EF* and 10.4 to 15.8 *CF*) compared to those (− 0.07 to 0.68 *I*_*geo*_, 2.3 to 10.7 *EF* and 1.5 to 2.5 *CF*) of Nakhonsawan Province.

**Table 1:** Pollution index of PTEs in soil samples near open waste bins and landfills

Element	Tak Province									Nakhonsawan Province					
	Pha De			Mae Pa			Ban Makluea			Panlan			Khaothong		
	*I*_*geo*_	*EF*	*CF*	*I*_*geo*_	*EF*	*CF*	*I*_*geo*_	*EF*	*CF*	*I*_*geo*_	*EF*	*CF*	*I*_*geo*_	*EF*	*CF*
Al	− 3.64	0.27	0.12	− 3.59	0.16	0.13	− 3.07	0.76	0.18	− 1.70	0.79	0.46	− 3.27	0.24	0.16
Cd	3.37	35.38	15.82	2.44	13.18	10.37	0.49	10.68	2.54	0.68	4.26	2.48	− 0.07	2.30	1.49
Cr	− 1.99	0.85	0.38	− 2.45	0.35	0.28	− 2.57	1.06	0.25	− 1.44	0.95	0.56	− 0.65	1.49	0.96
Cu	− 2.62	0.67	0.30	− 2.64	0.32	0.25	− 1.29	2.58	0.61	− 0.62	1.67	0.97	− 1.54	0.80	0.52
Fe	− 1.78	1.00	0.45	− 0.96	1.00	0.79	− 2.29	1.30	0.31	− 1.21	1.11	0.65	− 1.65	0.74	0.48
Mn	− 2.42	0.63	0.28	− 1.72	0.60	0.47	− 2.35	1.24	0.30	− 1.86	0.71	0.42	− 2.20	0.51	0.33
Ni	− 1.74	1.01	0.45	− 2.20	0.42	0.33	− 1.94	1.64	0.39	− 0.96	1.33	0.77	− 1.37	0.95	0.62
Pb	− 1.60	1.11	0.50	− 1.69	0.59	0.47	− 1.69	1.95	0.46	− 1.01	1.31	0.77	− 2.04	0.57	0.37
Zn	0.43	4.55	2.03	− 1.13	0.87	0.68	3.35	64.07	15.25	3.31	25.57	14.90	4.34	47.12	30.43

The *I*_*geo*_ values for Cd, notably for locations near open waste bins, were ranked as heavily contaminated. According to *EF* (35.4) and *CF* (> 6) values, soil Cd in Pha De was ranked as very high enrichment and extremely high enrichment, respectively. Substantial *I*_*geo*_ (2.4), *EF* (13.2) and *CF* values (> 6) were also determined in Mae Pa, which indicates that Cd contamination in the landfill was influenced by anthropogenic sources. Furthermore, low *I*_*geo*_ values of Cd (< 1) were noted in the landfills at Nakhonsawan Province, indicating that Cd was not a major soil pollutant.

All sampling locations had *PLI* values > 1 (Table 2). The highest *PLI* value was recorded at the Khaothong site (1.5), while the other sites exhibited comparable values, ranging from 1.3 to 1.4.

**Table 2:** Pollution load index values for PTEs in soil samples near open waste bins and landfills

Study area	Pollution load index value
Khaothong	1.49 ± 0.01a
Panlan	1.41 ± 0.01ab
Ban Makluea	1.40 ± 0.02bc
Mae Pa	1.32 ± 0.09c
Pha De	1.40 ± 0.03bc

Values followed by the same letter are not significantly different (LSD: *p* < 0.05)

### Necrophagous Fly Species in the Study Locations

A total of 9,662 necrophagous flies were captured from all study locations, varying in species composition and abundance based upon location (Table 3). The majority of flies were collected at Panlan, followed by Ban Makluea, Khaothong, Mae Pa and Pha De. More than 60% of the captured flies were calliphorid (Calliphoridae), followed by muscid (Muscidae) and sarcophagid (Sarcophagidae), respectively. Taxonomically, all specimens belonged to 13 species of these three families (Calliphoridae, Muscidae and Sarcophagidae) (Fig. S1, Table 3). Panlan and Ban Makluea in Nakhonsawan Province had substantial species richness values of flies (11 species and 8 species, respectively), along with substantial abundance values (4,874 individuals and 3,221 individuals, respectively) (Table 4). Mae Pa and Pha De in Tak Province had similar species richness values (11 species and 8 species, respectively); however, these locations had markedly low abundance values (314 individuals and 309 individuals, respectively), which were lower by 10.3–15.8⋅ when compared to Panlan and Makluea. However, Khaothong in Nakhonsawan Province, the location having the lowest species richness value (5 species) and also Margalef’s richness index (0.584), had a higher abundance value when compared to the other locations in Tak Province, by more than 3.0-3.1⋅. Values were consistent regarding other indices across all subdistricts. Mae Pa had the highest values for Shannon-Wiener’s diversity, Simpson’s abundance and Pielou’s eveness; in contrast, Khaothong exhibited the highest value of Dominance, specifically for *Musca domestica* (Linnaeus).

**Table 3:** Frequency of occurrence (%FO) of captured fly species from study locations

Genus Species	Pha De				Mae Pa				Ban Makluea				Panlan				Khaothong				Total				% frequency of occurrence
	M	F	T	% abund	M	F	T	% abund	M	F	T	% abund	M	F	T	% abund	M	F	T	% abund	M	F	T	% abund	
Family Calliphoridae																									
*Chrysomya megacephala*	37	121	158	51.13	54	69	123	39.17	705	904	1609	49.95	541	1628	2169	44.50	41	81	122	12.92	1378	2803	4181	43.27	100
*Chrysomya rufifacies*	16	19	35	11.33	12	23	35	11.15	335	628	963	29.90	367	597	964	19.78	16	34	50	5.30	746	1301	2047	21.19	100
Family Muscidae																									
*Atherigona orientalis*	0	0	0	0.00	0	0	0	0.00	2	0	2	0.06	5	0	5	0.10	0	0	0	0.00	7	0	7	0.07	40
*Atherigona* spp.	0	8	8	2.59	0	17	17	5.41	0	25	25	0.78	0	187	187	3.84	0	13	13	1.38	0	250	250	2.59	100
*Hydrotaea chalcogaster*	0	0	0	0.00	0	0	0	0.00	0	0	0	0.00	5	7	12	0.25	0	0	0	0.00	5	7	12	0.12	20
*Musca domestica*	8	38	46	14.89	26	38	64	20.38	143	367	510	15.83	428	982	1410	28.93	177	562	739	78.28	782	1987	2769	28.66	100
*Musca sorbens*	0	0	0	0.00	0	6	6	1.91	18	50	68	2.11	3	11	14	0.29	0	0	0	0.00	21	67	88	0.91	60
Family Sarcophagidae																									
*Boetcherisca peregrina*	1	0	1	0.32	6	0	6	1.91	0	0	0	0.0	4	0	4	0.08	0	0	0	0.00	11	0	11	0.11	60
*Liopygia ruficornis*	0	0	0	0.00	5	0	5	1.59	0	0	0	0.0	4	0	4	0.08	0	0	0	0.00	9	0	9	0.09	40
*Parasarcophaga dux*	5	0	5	1.62	6	0	6	1.91	4	0	4	0.12	7	0	7	0.14	0	0	0	0.00	22	0	22	0.23	80
*Parasarcophaga hirtipes*	1	0	1	0.32	1	0	1	0.32	0	0	0	0.00	0	0	0	0.00	0	0	0	0.00	2	0	2	0.02	40
*Parasarcophaga misera*	0	0	0	0.00	1	0	1	0.32	0	0	0	0.00	0	0	0	0.00	0	0	0	0.00	1	0	1	0.01	20
*Parasarcophaga* spp.	0	55	55	17.80	0	50	50	15.92	0	40	40	1.24	0	98	98	2.01	0	20	20	2.12	0	263	263	2.72	100
Total	68	241	309	100.00	111	203	314	100.00	1207	2014	3221	100.00	1364	3510	4874	100.00	234	710	944	100.00	2984	6678	9662	100.00	

% abund: % abundance (total number of fly species / total number of all fly species) ⋅ 100, M: male, F: female, T: total, frequency of occurrence (%): (number of study locations where flies were present / number of all study locations) ⋅ 100; < 40% FO: rare, 40–69% FO: moderately found, and > 70% FO: commonly found

**Table 4:** Species richness and diversity index of captured flies from five study locations

Diversity index	Tak Province		Nakhonsawan Province			Total
	Pha De	Mae Pa	Ban Makluea	Panlan	Khaothong	
Abundance	309	314	3,221	4,874	944	9,662
Species richness	8	11	8	11	5	13
Shannon-Wiener’s diversity index (*H*′)	1.379	1.716	1.186	1.303	0.752	1.329
Margalef’s richness index (*D*)	1.221	1.739	0.867	1.178	0.584	1.308
Simpson’s abundance index (*D′*)	0.671	0.763	0.635	0.677	0.367	0.684
Pielou’s evenness index (*J*)	0.663	0.716	0.570	0.543	0.467	0.518
Dominance index (*Y*)	0.329	0.237	0.365	0.323	0.633	0.316

*Chrysomya megacephala* (Fabricius) and *Chrysomya rufifacies* (Macquart), a calliphorid fly (Diptera: Calliphoridae), were distributed evenly across all studied locations, representing 100% of frequency of occurrence. Another fly species, *Musca domestica* (Diptera: Muscidae), also had 100% frequency of occurrence but was observed predominantly in the Khaothong (> 78% abundance) subdistrict of Nakhonsawan province. *Musca domestica* and *Atherigona* spp. (Rondani) were among the most observed species of Muscidae and were identified in all study locations. There were also less dominant necrophagous muscid files, namely *Atherigona orientalis* (Schiner), *Hydrotaea chalchogaster* (Wiedemann) and *Musca sorbens* (Wiedemann) which were identified in the disturbed forest near Ban Makluea and Panlan areas in Nakhonsawan.

Females of sarcophagid fly species were identified in all study locations although taxonomic classification was limited due to the similar nature of segmented body structures and morphological characteristics under the Family ‘Scarcophagidae’. In case of male flies, five species were identified; *Parasarcophaga dux* (Thomson) predominated, followed by *Boetcherisca peregrina* (Robineau-Desvoidy), *Liopygia ruficornis* (Fabricius), *Parasarcophaga hirtipes* (Wiedemann) and *Parasarcophaga misera* (Walker), respectively.

Abundance and diversity indices of necrophagoues flies were a function of location (Table 4). Highest and lowest species richness were noted in Mae Pa (11 species with 314 individuals) and Khaothong (5 species with 944 individuals), respectively, which aligns with highest and lowest richness indices (*D* = 1.739 in Mae Pa and *D* = 0.584 in Khaothong). Panlan also had highest species richness (11) with 4,874 fly individuals; however, it did not attain highest *D* value (≈1.2). A similar trend was observed in Pha De subdistrict where both species richness (8) and index (*D* = 1.221) were comparable to those of other subdistricts. Substantial fly abundance and species richness may be associated with a lower *J* value, as evidenced by the lower value in Panlan (0.543) compared to Pha De (0.663) and Ban Makluea (0.570). The dominant population is more pronounced when species are distributed less evenly. *Musca domestica* was noted in highest number as a result of lowest *J* value (0.467) and highest *Y* value (0.633) at Khaothong,

The similarity test was analyzed for all necrophagous fly species based on study location, and a paired-wise similarity degree was demonstrated (Table 5). With the exception of two pairs (Khaothong and Panlan; Khaothong and Mae Pa) having very low *Cj* values of 0.385 and 0.454, all other pairs had *Cj* values > 0.5. Highest species similarity was observed between Mae Pa and Panlan (*Cj* = 0.846) where each location belonged to different and separate provinces.

**Table 5:** Similarity coefficient (*Cj*) value of captured flies from five study locations

Similarity index	Pha De	Mae Pa	Ban Makluea	Panlan	Khaothong
Pha De	1.0000	0.727	0.600	0.583	0.625
Mae Pa	–	1.000	0.545	0.846	0.454
Ban Makluea	–	–	1.000	0.800	0.625
Panlan	–	–	–	1.000	0.385
Khaothong	–	–	–	–	1.000

### Potentially Toxic Element Accumulation in Fly Tissues

**Fig. 3:**
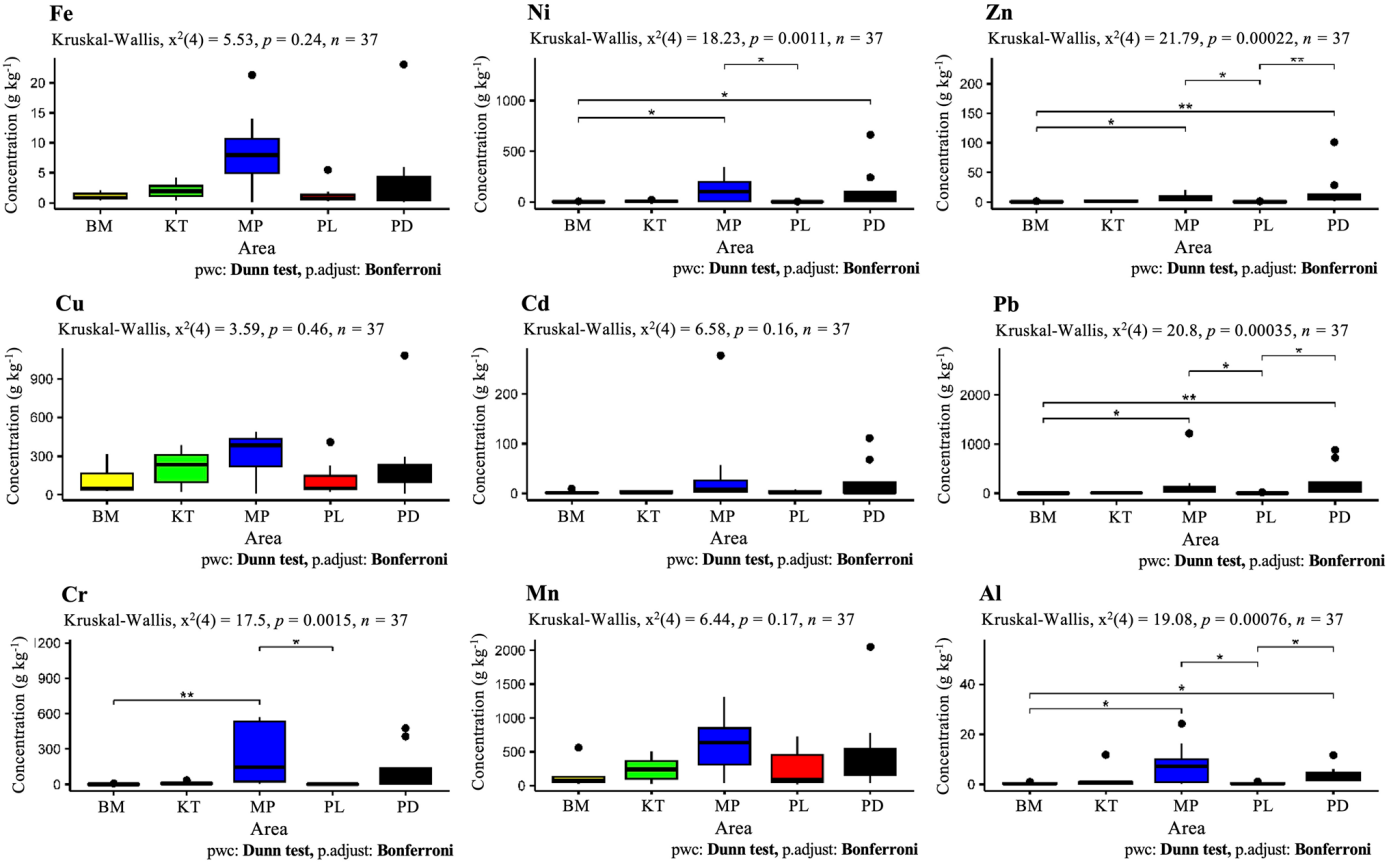
Boxplot from five sampling locations showing concentrations of each PTE in necrophagous fly tissue. *p* * < 0.05; *p* ** < 0.01; *p* *** < 0.001; dots represent outliers. BM: Ban Makluea, KT: Khaothong, MP: Mae Pa, PL: Panlan and PD: Pha De

Concentrations of accumulated toxic elements varied with study location in the order: Zn > Fe > Al > Mn > Cu > Pb > Cr > Ni > Cd (Table S4). Different fly species responded to PTE bioaccumulation at different concentrations (Fig. 3; Table S4). Zinc, Fe and Al were among the most accumulated elements in different fly species, ranging in concentration (g kg^−1^) by 0.14–101, 0.15-23 and 0.03–2.1, respectively. Conversely, Ni and Cd accumulated at lower quantities, in the ranges of 0.1-663.6 mg kg^−1^ and 0.2-277.7 mg kg^−1^, respectively.

The most abundant fly species in Tak Province accumulated substantial PTEs while flies captured from subdistricts of Nakhonsawan province accumulated lower concentrations. Flies in Pha De (Tak province) experienced relatively or significantly highest bioaccumulation of Cu (1,082 mg kg^−1^), Mn (2,051 mg kg^−1^), Ni (664 mg kg^−1^), and Zn (101 g kg^−1^) while those in Mae Pa had highest concentrations of Al (8.1 g kg^−1^), Cd (278 mg kg^−1^), Cr (571 mg kg^−1^), Fe (9.6 g kg^−1^) and Pb (1,214 mg kg^−1^), respectively. As described above, Nakhonsawan subdistricts represented lowest concentrations of bioaccumulated metals (except for 11 mg kg^−1^ Cu and 0.2 g kg^−1^ Fe, which were lowest in Pha De of Tak province), including Al (0.1 g kg^−1^), Cd (0.2 mg kg^−1^), Cr (0.3 mg kg^−1^), Mn (19 mg kg^−1^), Ni (0.1 mg kg^−1^), Pb (0.3 mg kg^−1^) and Zn (0.2 g kg^−1^). Two necrophagous fly species, *M. domestica* and *C. rufifacies*, accumulated greatest quantities of PTEs; highest concentrations of Cu, Mn, Ni and Zn accumulated in both male and female *M. domestica* flies, and lowest concentrations of Cd, Mn and Zn were detected in male *C. rufifacies*.

**Fig. 4:**
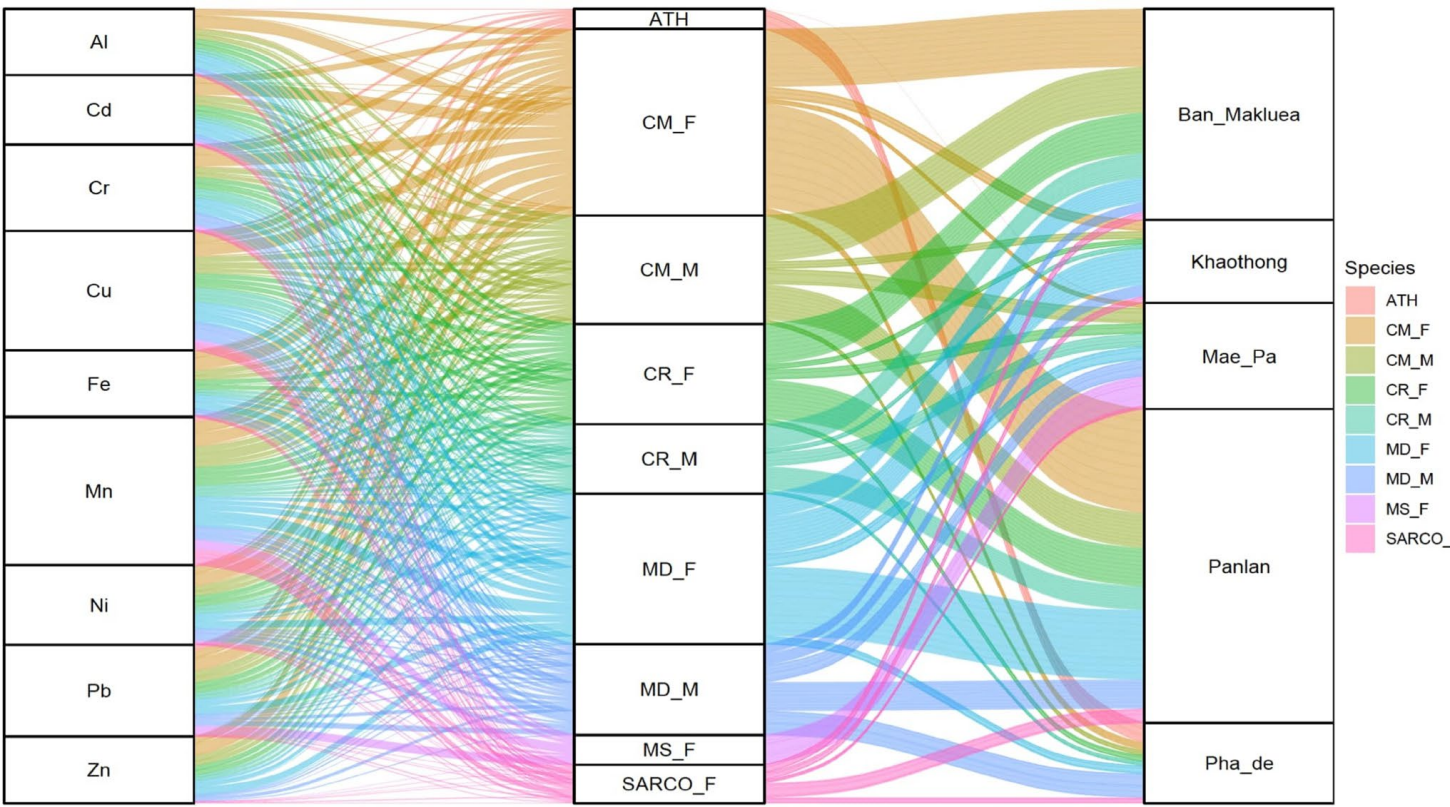
Alluvial plot illustrating the network of most abundant fly species interactions with accumulated PTEs with respect to habitat (subdistrict) in Tak and Nakhonsawan provinces. Each color represents a specific fly species. CM: *Chrysomya megacephala*, CR: *Chrysomya rufifacies*, MD: *Musca domestica*, MS: *Musca sorbens*, ATH: *Atherigona* spp., SARCO: *Sarcophaga* spp., BM: Ban Makluea, KT: Khaothong, MP: Mae Pa, PL: Panlan and PD: Pha De, M: Male, F: Female

**Fig. 5:**
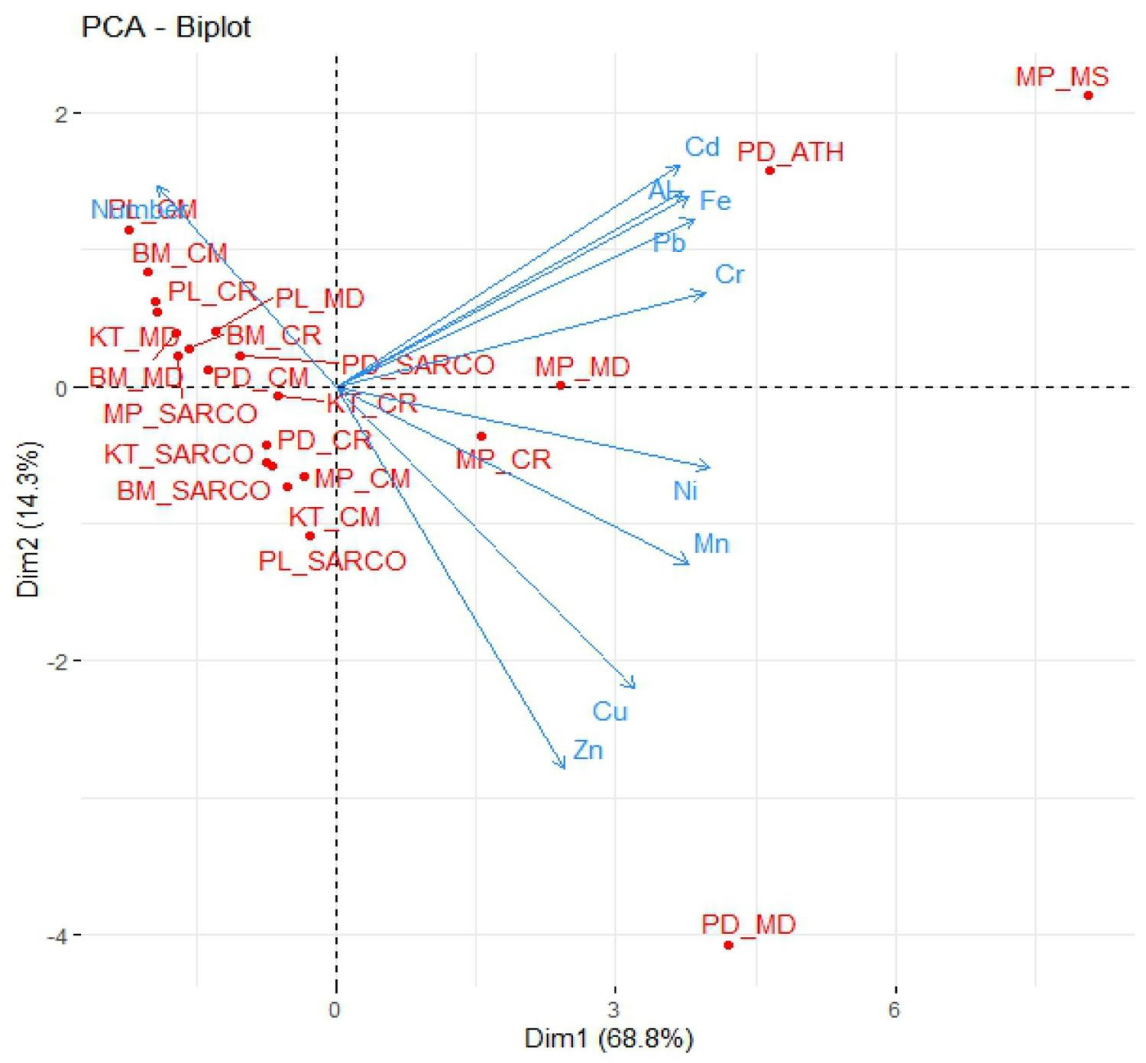
Principal component analysis between PTE concentration in each fly species and sampling location. CM: *Chrysomya megacephala*, CR: *Chrysomya rufifacies*, MD: *Musca domestica*, MS: *Musca sorbens*, ATH: *Atherigona* spp., SARCO: *Sarcophaga* spp., BM: Ban Makluea, KT: Khaothong, MP: Mae Pa, PL: Panlan and PD: Pha De

Different fly species were distributed throughout the selected subdistricts of each province and their abundances corresponded with accumulated PTEs in the respective habitats. The network flow was displayed in an alluvial plot (Fig. 4) depicting networks of variables under the different groups, e.g., fly species and accumulated PTEs, with respect to location. The most abundant fly species was *C. megacephala* and the habitat with highest species richness was Panlan, followed by Ban Makluea (Fig. 4). To visualize how the abundance patterns of necrophagous fly species that accumulated PTEs correlated with accumulated toxic elements within the specific habitat (subdistrict), principal component analysis (PCA) was conducted (Fig. 5). The abundance of *Atherigona* spp. and *M. sorbens* were observed in Pha De and Mae Pa where Cd, Al, Fe, Pb, and Cr were primarily distributed. Another clear separation by *M. domestica* abundance was in Pha De subdistrict where Zn, Cu, Mn and Ni were occurred in high concentrations. Abundances of certain fly species were associated with areas enriched in specific elements; for example, *C. rufifacies* were abundant in the presence of Mn and Ni enrichment in the soil in Mae Pa, while *Sarcophaga* spp. and *M. domestica* flies were abundant in Cr-enriched area of Mae Pa.

## Discussion

Potentially toxic metals have been discovered in numerous municipal landfill leachates from solid waste disposal sites and hazardous waste landfills (Essien et al. 2022). In the current study, Fe and Al were greater than those of other elements measured in the study locations. These elements are abundant in the earth’s crust, which is consistent with the findings of previous studies (Neenu and Karthika 2019; Printarakul and Meeinkuirt 2022). The high soil Cd concentration detected at Pha De (1.6 mg kg^−1^) compared to all other locations, indicates anthropogenic contamination, as soil Cd content should not exceed 1 mg kg^−1^ for agricultural soil established by National Soil Quality Standardization Technical Committee of China (Xu et al. 2015). Zinc concentrations in landfills in Nakhonsawan province exceeded threshold levels, but concentrations of other PTEs did not exceed threshold values for mineral soil (Neenu and Karthika 2019). Elevated Cd and Zn concentrations in water and soil in the Pha De subdistrict are mainly linked to long-term discharges from mining activities into nearby streams and agricultural areas over the past three decades (Weeraprapan et al. 2015). Furthermore, Cu and Ni contents were within the permissible range of 100 mg kg^−1^ (as established for Canada, Austria, and Poland) for all study locations (Kabata-Pendias and Mukherjee 2007).

Elevated pollution index values include the *I*_*geo*_ values for Cd, notably in the location near open waste bins, which were ranked as heavily contaminated. Research has not been conducted previously on soils in the current study location, which is a populated area. Previous studies have assessed levels of Cd and Zn in biota and in ecosystems in the agricultural zone of the Mae Tao River Basin (Saengwilai and Meeinkuirt 2021). The hypothesis of Cd and Zn dispersion within these areas and neighboring locations from mining remains valid, even though the Zn mine ceased operating in 2016 (Nakbanpote et al. 2018).

Other elements i.e., Al, Cr, Cu, Fe, Mn, Ni and Pb typically had *I*_*geo*_ values < 0, *EF* values < 2 and *CF* values < 1, indicating that they were not the primary source of contamination in any study location. Even at low concentrations, Cd and Zn can impart hazardous effects on biota; hence, it is important to monitor their concentrations in and near landfills.

The *EF* index indicates that municipal waste is the primary source of Zn in landfills in Nakhon Sawan Province. In addition, the pollution index revealed that Zn was not a major soil pollutant in Tak Province, including the location near open waste bins. Substantial evidence indicates that municipal solid waste components, including cleaning products, cosmetics and shampoos, paints and pigments, and lubricants, increase Zn contamination in landfills (Ngole and Ekosse 2012). The zinc mine in Mae Sot District is widely recognized as the primary source of Zn and Cd in the Mae Tao River Basin including agricultural areas (Saengwilai and Meeinkuirt 2021). However, metal accumulation in some locations might be the result of introducing soil, sand and/or other material from outside the area to cover the contaminated soil for community building projects.

The observed *PLI* values suggest widespread PTE contamination in soil, particularly at landfill sites. This highlights the need for routine groundwater monitoring in these areas, as there is a potential risk of PTE-contaminated leachate infiltrating the surrounding environment. Such contamination can lead to significant adverse effects on both human health and environmental quality (Chen et al. 2020; Talalaj 2015). Notably, the Khaothong site, with the highest *PLI* value, may be influenced by elevated soil zinc concentrations, underscoring the importance of specific element monitoring in relation to *PLI* variations (Kükrer et al. 2014).

*Chrysomya megacephala* and *C. rufifacies* (Macquart), a calliphorid fly (Diptera: Calliphoridae), were found in all study locations, representing 100% of frequency of occurrence. However, *M. domestica* (Diptera: Muscidae) predominated in Khaothong. *Chrysomya megacephala* and *M. domestica* are important necrophagous species that are referred to as synanthropic species, as they live in proximity to and benefit from humans (Chaiwong et al. 2012; Moophayak et al. 2014). The housefly *M. domestica* is the best-known and most widespread fly present around human dwellings worldwide as compared to *C. megacephala*. Because *M. domestica* are highly adapted to human settings including harsh environments, they have relatively high biological fitness, resulting in exceptional survival and reproductive success (Sukontason et al. 2018).

Many reports indicate that environmental factors such as habitat, temperature, and seasonal adaptation affect the oviposition habits and activities of flesh flies and necrophagous blow flies (Lutz et al. 2022). Habitat characteristics are among the most important factors influencing the presence of necrophagous flies at locations suitable for dwelling. In this study, the environmental variables in Pha De and Mae Pa may be similar as they are situated at similar elevations above sea level while being located far from the landfills at Nakhonsawan. To some extent, temperature and wind may also comprise variables that affect fly communities in each location. According to several studies, *C. rufifacies* prefers open locations having warm temperatures rather than crowded, damp locations, as evidenced by its higher abundance in open pastures as opposed to forested habitats (Cammack and Nelder 2010; Palmer 1980). However, an open paddy field might not be a favorable habitat for this necrophagous species because numerous farming activities take place during the crop cycle that may prove disruptive. Klong-klaew et al. (2014) reported that *C. rufifacies* prefers to reside in disturbed mixed deciduous forests and lowland villages rather than paddy fields. The Khaothong landfill is also surrounded by paddy fields; *C. rufifacies* had a lower % abundance compared to the other locations by a factor of 2.1–5.6⋅. *Chrysomya rufifacies* was captured primarily in intact forests (i.e., tall trees), although some disturbed areas also occur within these forests. It is noteworthy that, to some extent, fly communities recorded higher species richness in the forest than in lowland villages or urban areas (Klong-klaew et al. 2018).

In this study, *M. domestica* and *Atherigona* spp. (Rondani) comprised the predominant species of muscid fly and were identified in all study locations. *Musca domestica* are distributed primarily in residential areas and feed on fecal matter and wet waste from fresh food markets (Chaiwong et al. 2012). They have been found in other human-influenced environments such as poultry farms, agricultural fields, and disturbed deciduous forests (Sukontason et al. 2018). Small numbers of the other three necrophagous muscid species (*Atherigona orientalis* (Schiner), *Hydrotaea chalcogaster* (Wiedemann), and *Musca sorbens* (Wiedemann) were identified in the disturbed forest near Ban Makluea and Panlan. *Musca sorbens* is attracted by carboxylic acid, cresol, and indole compounds from rotting proteinaceous foods, livestock, and human feces (Robinson et al. 2021). Other muscid species like *Musca ventrosa* (Wiedemann) prefer a tree-rich habitat, as demonstrated by reports finding it in orchards and forest habitats (Klong-klaew et al. 2020). The adult *A. orientalis* is attracted to decaying plant material, dung and carrion of vertebrates, where they lay their eggs. After hatching, the larvae act as scavengers or predators that feed on decaying organic matter (Cabrera-Cánoves et al. 2019). Almost 300 species of larval necrophagous flies, the majority of which are members of the Calliphoridae, Muscidae, and Sarcophagidae families, occur in Thailand around decaying plant and animal material (Bunchu 2012; Kurahashi and Samerjai 2018; Moophayak et al. 2011; Tumrasvin and Shinonaga 1977, 1978, 1982). *Atherigona orientalis* are widely distributed in urban areas in Thailand at elevations of 284 to 805 m above sea level (Moophayak et al. 2011). *Hydrotaea chalcogaster* is widely distributed in the warm tropics. This species is an important flesh fly that is employed to aid in forensic investigations due to its attraction to human corpses during the butyric and caseic fermentation phases (Verçosa et al. 2018).

Females of sarcophagid fly species were identified in all study locations, but species identification was limited due to similarities in morphological characteristics. According to Bänziger and Pape (2004), larviposition is the key factor that results in more sarcophagid females than males visiting feces and cadavers. In this study, male flies of five species were identified. Males of *Parasarcophaga dux* (Thomson) were predominant, followed by *Boetcherisca peregrina* (Robineau-Desvoidy), *Liopygia ruficornis* (Fabricius), *Parasarcophaga hirtipes* (Wiedemann) and *Parasarcophaga misera* (Walker). All species were found in broiler carcasses according to an investigation conducted in Nakhon Sawan (Moophayak et al. 2017), while *P. misera* predominated near feces (Sukontason et al. 2010). Little is known about *P. dux*, *B. peregrina*, and *L. ruficornis* in field environments. These fly species can be employed for forensic investigations, despite the fact that they are typically found on animal carcasses (Bänziger and Pape 2004; Sukontason et al. 2010). Seasonal period affected the immature development of the sarcophagid fly – a short period of growth was noted during the dry season, when temperatures were elevated. As a result, more adult flies were present during this period than at other seasonal periods, and they might be mating (Sukontason et al. 2010).

The lower *D* value in species richness analysis indicates lower diversity of organisms in the terrestrial ecosystem (Meng et al. 2020). In the current study, the lowest *D* value for species richness in Khaothong, which may be influenced by agriculture and other anthropogenic activities within the habitat (Amin et al. 2024). Substantial fly abundance and species richness with lower *J*-values may be explained by the elevated numbers of certain common or rare fly species, resulting in a decreased measure of species evenness (Magurran 1998). Furthermore, *D′* and *J* had similar trends; however, *D′* values were weighted toward abundance of the most common species rather than species richness (Pollard et al. 1995). Environmental factors (i.e., temperature, wind direction) and food supply, particularly in landfills, are key influences on their abundance, species richness and species diversity (Howard 2001; Lole 2005; MacDonald et al. 2017).

The degree of similarity of the necrophagous fly species in all study locations is demonstrated in Table 5. With the exception of Khaothong and Panlan and Khaothong and Mae Pa, whose *Cj* values were very low (0.385 and 0.454, respectively), Mae Pa and Panlan had the highest *Cj* value (0.846), and all other pairs had values higher than 0.500. In this study, it was evident that a high degree of similarity exists between each pair of locations due to the presence of *L. ruficornis* in Panlan and Mae Pa, *M. sorbens* and *(A) orientalis* in Panlan and Ban Makluea, and *(B) peregrina*, *P. hirtipes* and *P. misera* in Pha De and Mae Pa. The similar fly communities in all study locations, particularly in landfills, may be linked with optimal environmental conditions (i.e., waste composition, waste moisture content, optimal climate).

Leachate generation from landfills is regarded as a key source of PTEs that contaminates soil and water resources and ultimately exposes biota in the food chain. Long-term exposure to PTEs has the potential to harm health of biota and alter community structure. Species richness and abundance of biota can be taken into account in the diversity index to monitor environmental pollution in contaminated areas. The markedly low diversity index values, particularly *H՛* (< 2) and *D* (< 2) in all study locations are rated as severe to heavy pollution (Zhu et al. 2021), whereas *J* values < 0.75 suggest that fly species are categorized as depressed community across all study locations, resulting in low species richness and abundance (Krebs 1989). As shown by several studies, environmental factors, particularly elevated temperature and low precipitation during the dry season, may also impart a considerable impact on fly communities (Godwin et al. 2017; Taylor et al. 2017).

Male flies feed primarily on pollen or nectar of certain flowering plants, some of which may accumulate PTEs from municipal waste leachates or other sources. Moreover, the marked buildup of PTEs, particularly Zn and Cd in male *M. domestica* at Pha De, may have been influenced by the Zn- and Cd-polluted Mae Tao River Basin, which now contains substantial PTEs in soil (Roongtanakiat and Sanoh 2015). Male flies in Mae Pa and Pha De of Tak Province may serve as bioindicators, as they accumulated substantial concentrations of PTEs. According to several investigations, food waste and animal manures in exposed municipal wastes and landfills, both of which serve as fly substrate, contain substantial PTE concentrations. Such substrates likely increase the degree of PTE accumulation in fly tissue, which is then transferred to consumers higher in food webs (Diener et al. 2015a; Gold et al. 2018).

It has been reported that insect species diversity and richness fluctuate or shift due to type and quality of habitat; this may include environmental pollution (e.g., from PTEs) (Saberi Pour et al. 2024). Understanding and documenting insect adaptation to changing environments is crucial for effective conservation efforts. The substantial abundances of *M. sorbens* in Mae Pa and *Atherigona* spp. in Pha De in the presence of Cd, Al, Fe, Pd, and Cr via PCA analysis suggest that these species are candidate bioindicators of respective elements with respect to location (Soliman et al. 2024). Necrophagous flies should be abundant, easy to sample, and respond rapidly to environmental change in order to meet the criteria for an effective bioindicator of anthropogenic effects (Amat and Medina 2021). *Atherigona* spp. displayed low abundance when compared to other species. They are difficult to capture in field settings, so a more appropriate bait may be needed to attract greater numbers of flies, including male flies to aid in identification. This species in Thailand has been reported in a few articles; little is known, therefore, as regards their biology and potential vulnerability to environmental change.

Insects have long been used as bioindicators because they have a strong relationship for harsh settings, especially locations contaminated with PTEs (Azam et al. 2015). Several studies have found that PTEs impart deleterious effects on *M. domestica* as determined by decrease in body weight, developmental abnormalities, lower reproduction, decreased hatchability and survival rate of the larvae, extension of developmental duration and changes in oviposition dynamics (Haq 2013; Tylko et al. 2006). Altered morphological and physiological traits by *M. domestica* is evidence that PTEs have harmful effects when assessed in mesocosm settings. *Musca domestica* typically feeds on exposed municipal wastes that may have accumulated PTEs. This fly species is a promising bioindicator for monitoring PTE pollution because it is typically found in varied municipal waste types worldwide (Haq 2013).

## Conclusions

The current study provides an advanced understanding of necrophagous fly diversity and species richness in two provinces in Thailand, evaluating interactions with PTEs in selected habitats. Variation in fly species abundance among the two provinces (Tak and Nakhonsawan) contributes to our understanding of behavioral patterns, ecological dynamics and habitat preference of each species as influenced by specific PTEs. *Musca* spp. and *Chrysomya* spp. are among the most abundant among 13 detected species across the studied habitats, of which *Musca* spp., especially *M. domestica*, showed significant bioaccumulation capacity of certain elements (e.g., Cr, Cu, Mn, Ni and Zn). This species is thus proposed as a potential bioindicator of those elements, depending on habitat. *Chrysomya* spp., particularly *C. rufifacies*, can be serve as a bioindicator due to its abundance in the presence of Al, Cr, Ni, and Zn in Nakhonsawan province. Our findings highlight fly species abundance and bioaccumulation of PTEs at different concentrations with respect to selected habitats of Thailand; such data has not been previously reported.

## Supplementary Information

Below is the link to the electronic supplementary material.

Supplementary Material 1

## Data Availability

Data will be made available on request.
