# Supplementary material for: Accumulation of Potentially Toxic Elements and Bioindicator Potential of Necrophagous Flies in Exposed Municipal Wastes
Source: Arch Environ Contam Toxicol. 2026 Mar 23;90(3):17. doi: 10.1007/s00244-026-01186-5 (PMC13009087; doi:10.1007/s00244-026-01186-5)
Supplement: Supplementary file 1 — Supplementary Material 1 [file 244_2026_1186_MOESM1_ESM.docx]

**
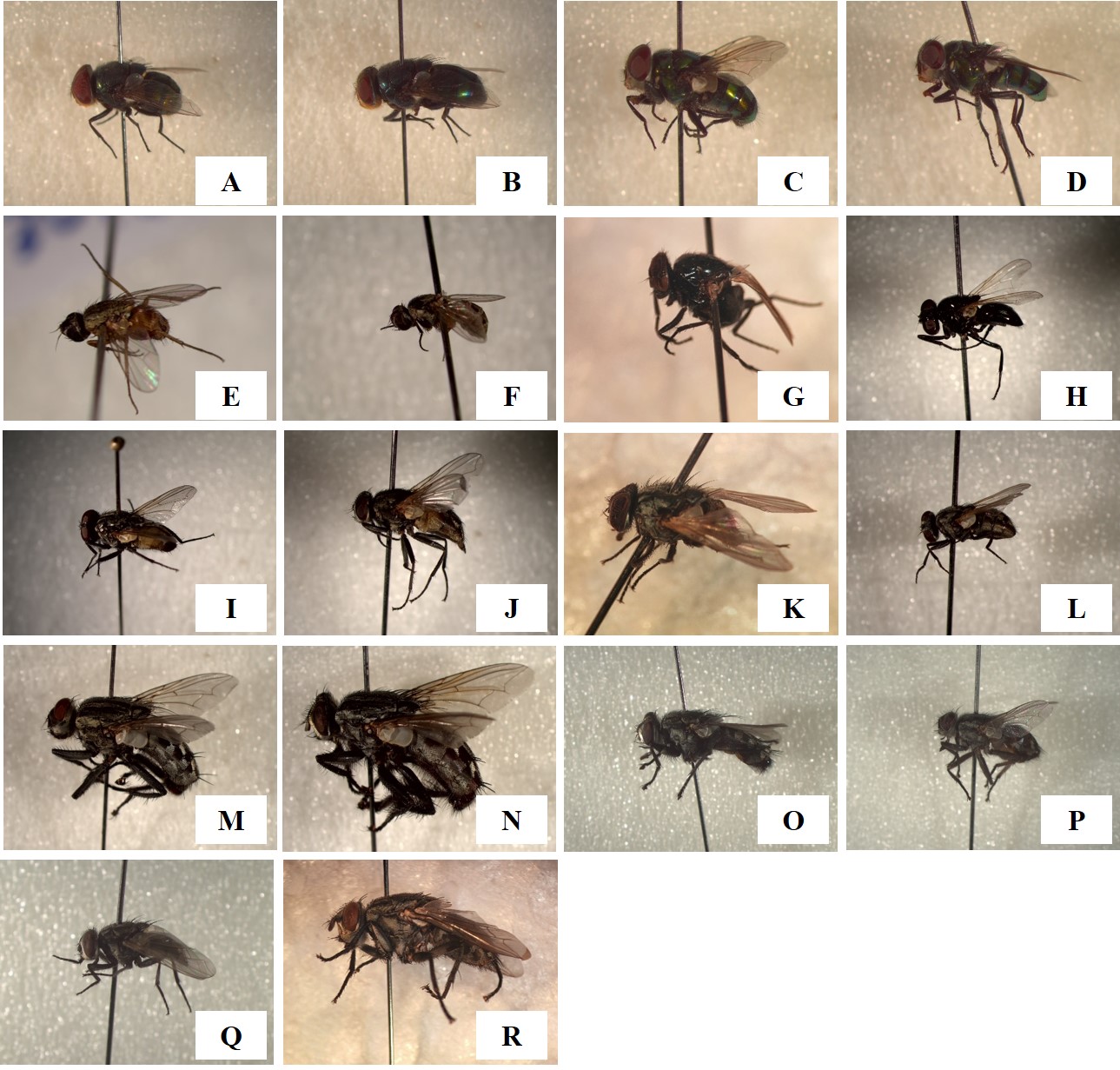
**

**Fig. S1.** Lateral view of captured flies. *Chrysomya megacephala* (Fabricius) male (CM-M) (A) and female (CM-F) (B); *Chrysomya rufifacies* (Macquart) male (CR-M) (C) and female (CR-F) (D); *Atherigona orientalis* (Schiner) male (E); *Atherigona* spp. Rondani female (Ath) (F); *Hydrotaea chalcogaster* (Wiedemann) male (G) and female (H); *Musca domestica* (Linnaeus) male (MD-M) (I) and female (MD-F) (J); *Musca sorbens* (Wiedemann) male (K) and female (MS-F) (L); *Boetcherisca peregrina* (Robineau-Desvoidy) male (M); *Liopygia ruficornis* (Fabricius) male (N); *Parasarcophaga dux* (Thomson) male (O); *Parasarcophaga hirtipes* (Wiedemann) male (P); *Parasarcophaga misera* (Walker) male (Q); and *Sarcophaga* spp. (Johnston and Tiegs) female (SARCO-F) (R).

**Table S1** GPS coordinates of the five study locations in Tak and Nakhon Sawan Provinces, Thailand

| **Study location** | **GPS coordinates** | **Waste accumulation** | **Location** |
| --- | --- | --- | --- |
| **Pha De** | 16° 40’ 32.95” N, 98° 37’ 46.91”E, 256 msl. | Open waste bins | Pha De subdistrict, Mae Sot district, Tak Province |
| **Mae Pa** | 16° 43’ 53.35” N, 98° 31’ 40.06” E, 200 msl. | Landfill | Mae Pa subdistrict, Mae Sot district, Tak Province |
| **Ban Makluea** | 15° 48’ 58.95” N, 100° 7’ 48.35” E, 28 msl. | Landfill | Ban Makluea subdistrict, Muang district, Nakhon Sawan Province |
| **Panlan** | 15° 50’ 42.79” N, 100° 15’ 47.46” E, 27 msl. | Landfill | Panlan subdistrict, Chumsaeng district, Nakhon Sawan Province |
| **Khaothong** | 15° 32’ 52.13” N, 100° 11’ 51.61” E, 70 msl. | Landfill | Khaothong subdistrict, Phayuha Kiri district, Nakhon Sawan Province |

**Table S2** Typical instrumental parameters (AAnalyst 600, PerkinElmer^®^)

| Heavy metal | Wavelength (nm) | Lamp current (mA) | Slit width (nm) | Flame type/Mode |
| --- | --- | --- | --- | --- |
| Cadmium | 228.8 | 4.0 | 0.7 | Air-acetylene (Flame) |
| Copper | 324.8 | 5.0 | 0.7 | Air-acetylene (Flame) |
| Aluminum | 309.3 | 5.0 | 0.2 | Air-acetylene (Flame) |
| Iron | 248.3 | 5.0 | 0.2 | Air-acetylene (Flame) |
| Manganese | 279.5 | 5.0 | 0.2 | Air-acetylene (Flame) |
| Zinc | 213.9 | 10.0 | 0.7 | Air-acetylene (Flame) |
| Chromium | 357.9 | 7.0 | 0.7 | Air-acetylene (Flame) |
| Nickle | 232.0 | 5.0 | 0.2 | Air-acetylene (Flame) |
| Lead | 283.3 | 5.0 | 0.7 | Air-acetylene (Flame) |

**Table S3** PTE concentrations in soil at five study locations (*n* = 3)

| Element | Unit | Tak Province | |  | Nakhon Sawan Province | | |
| --- | --- | --- | --- | --- | --- | --- | --- |
|  |  | Pha De | Mae Pa |  | Ban Makluea | Panlan | Khaothong |
| Al | g kg^-1^ | 9.7±0.8b | 10±1.3b |  | 9.9±6.0b | 29±7.1a | 10±4.4b |
| Cd | mg kg^-1^ | 1.6±0.4a | 1.0±0.8b |  | 0.1±0.1c | 0.1±0.1c | 0.1±0.1c |
| Cr | mg kg^-1^ | 13±1.7b | 9.6±0.8b |  | 11±6.8b | 17±4.2b | 33±13a |
| Cu | mg kg^-1^ | 7.4±4.5cd | 6.2±1.9d |  | 13±3.5bc | 23±4.6a | 18±7.1b |
| Fe | g kg^-1^ | 16±3.8ab | 28±6.6a |  | 8.3±3.3b | 20±5.7a | 23±14a |
| Mn | mg kg^-1^ | 169±20ab | 282±79a |  | 215±71ab | 226±86a | 156±60b |
| Ni | mg kg^-1^ | 9.1±1.3b | 6.6±0.7b |  | 9.8±8.1b | 16±4.9a | 7.9±4.3b |
| Pb | mg kg^-1^ | 9.9±0.8a | 9.4±1.3a |  | 9.0±5.2a | 10±4.5a | 6.8±3.9a |
| Zn | mg kg^-1^ | 144±18b | 49±2.8b |  | 1302±580a | 1571±729a | 1832±702a |

For each metal in a specific location, values followed by the same letter are not significantly different (LSD: *p* < 0.05)

*Al* = aluminum, *Cd* = cadmium, *Cr* = chromium, *Cu* = copper, *Fe* = iron, *Mn* = manganese, *Ni* = nickel, *Pb* = lead, *Zn* = zinc

**Table S4** PTE concentrations in necrophagous flies at the five study locations (*n* = 3)

| **Location** | **Species** |  | | | | | | | | |
| --- | --- | --- | --- | --- | --- | --- | --- | --- | --- | --- |
|  |  | **Al (g kg^-1^)** | **Cd (mg kg^-1^)** | **Cr (mg kg^-1^)** | **Cu (mg kg^-1^)** | **Fe (g kg^-1^)** | **Mn (mg kg^-1^)** | **Ni (mg kg^-1^)** | **Pb (mg kg^-1^)** | **Zn (g kg^-1^)** |
| Pha De | CM-M | 3.4±2.2bc | 1.3±1.0c | 3.6±3.1b | 124±105b | 0.2±0.1e | 173±161b | 2.1±1.0c | 65±29b | 1.0±0.4b |
|  | CM-F | 1.5±1.4c | 0.7±0.5c | 0.9±0.6b | 25±22b | 0.2±0.0e | 35±17b | 2.3±1.8c | 13±12b | 4.3±3.3b |
|  | CR-M | 1.8±1.6c | 1.5±1.3c | 11±10b | 207±203b | 1.3±0.8cde | 124±98b | 29±16bc | 38±24b | 8.8±6.9b |
|  | CR-F | 1.2±0.5c | 1.3±1.1c | 44±26b | 162±135b | 3.0±1.1cd | 344±282b | 52±36bc | 50±40b | 4.6±1.6b |
|  | MD-M | 4.1±1.3bc | 68±31b | 477±288a | 1082±932a | 6.0±3.6b | 2051±1717a | 664±399a | 727±598a | 101±951a |
|  | MD-F | 4.1±3.3bc | 6.2±5.1c | 7.7±3.5b | 213±142b | 3.8±2.2bc | 470±267b | 38±16bc | 51±11b | 28±9.8b |
|  | ATH-F | 12±3.7a | 111±35a | 407±103a | 297±43b | 23±3.4a | 783±322b | 241±44b | 886±1.8a | 7.9±1.5b |
|  | SARCO-F | 6.3±2.8b | 2.6±1.6c | 11±6.0b | 11±0.5b | 0.4±0.0de | 236±156b | 10±7.3c | 19±15b | 5.4±4.2b |
| Mae Pa | CM-M | 0.6±0.5de | 4.2±2.7d | 30±29bcd | 424±270ab | 9.6±4.8c | 979±570ab | 6.3±3.7d | 212±151b | 20±16a |
|  | CM-F | 1.1±1.0de | 0.5±0.3d | 4.5±3.4cd | 16±8.1c | 0.3±0.2d | 53±7.6de | 5.8±4.2d | 6.4±1.9e | 0.9±0.3c |
|  | CR-M | 8.1±2.3c | 5.7±4.7cd | 526±53a | 484±235a | 8.7±2.1c | 396±201cd | 172±128bc | 109±45cd | 7.7±2.5bc |
|  | CR-F | 7.2±1.3cd | 12±7.8cd | 165±42b | 287±17b | 6.5±2.0c | 597±301bc | 111±79cd | 51±25cde | 5.8±2.4c |
|  | MD-M | 16±10b | 57±24b | 571±259a | 419±190ab | 14±3.8b | 678±361bc | 265±222ab | 114±26c | 6.4±4.4c |
|  | MD-F | 7.6±5.7c | 16±8.0c | 125±62bc | 348±45ab | 7.2±0.4c | 807±170b | 90±98cd | 46±25de | 3.6±1.7c |
|  | MS-F | 24±4.3a | 278±2.9a | 556±68a | 458±102ab | 21±5.7a | 1314±355a | 345±44a | 1214±15a | 17±5.7ab |
|  | SARCO-F | 0.3±0.2e | 0.8±0.4d | 3.7±1.9d | 11±4.5c | 0.2±0.2d | 37±18e | 4.0±2.7d | 4.6±1.7e | 0.8±0.3c |
| Ban Makluea | CM-M | 0.3±0.1b | 0.7±0.6b | 0.7±0.5c | 88±50b | 0.8±0.6b | 71±53b | 0.9±0.6c | 1.4±0.2b | 0.3±0.3b |
|  | CM-F | 0.2±0.1b | 1.1±0.6b | 0.6±0.3c | 48±35b | 0.6±0.3b | 45±19b | 0.7±0.4c | 0.6±0.5b | 0.3±0.2b |
|  | CR-M | 1.0±0.1a | 1.2±0.2b | 8.0±0.6a* | 316±10a | 2.1±0.2a | 126±23b | 6.9±0.4a | 9.1±2.2a | 1.4±0.3a |
|  | CR-F | 0.1±0.0b | 0.7±0.4b | 0.5±0.1c | 31±17b | 0.4±0.2b | 30±12b | 0.5±0.1c | 0.5±0.2b | 0.3±0.1b |
|  | MD-M | 0.3±0.2b | 0.6±0.3b | 0.8±0.2c | 51±24b | 0.9±0.2b | 51±22b | 1.0±0.3c | 0.7±0.5b | 0.3±0.2b |
|  | MD-F | 0.2±0.1b | 2.4±0.4b | 0.6±0.4c | 31±22b | 1.2±0.4ab | 126±97b | 0.6±0.5c | 1.7±0.5b | 0.3±0.2b |
|  | SARCO-F | 0.7±0.6a | 9.5±6.4a | 3.2±2.5b | 246±194a | 1.8±1.4a | 560±421a | 2.9±2.4b | 8.8±5.1a | 0.7±0.6b |
| Panlan | CM-M | 0.3±0.1c | 0.7±0.3c | 1.3±1.0c | 41±19c | 0.8±0.4c | 51±27bc | 1.3±0.9c | 0.6±0.4c | 0.3±0.1c |
|  | CM-F | 0.2±0.1c | 0.4±0.2c | 0.6±0.3c | 46±20c | 0.7±0.5c | 92±61bc | 0.8±0.5c | 0.3±0.2c | 0.3±0.2c |
|  | CR-M | 0.1±0.0c | 2.4±1.6b | 0.9±0.1c | 25±4.3c | 0.3±0.2c | 19±8.8c | 0.9±0.4c | 1.1±0.9c | 0.2±0.0c |
|  | CR-F | 0.2±0.1c | 0.4±0.2c | 0.8±0.4c | 47±29c | 0.8±0.4c | 45±29c | 0.8±0.4c | 1.5±1.2c | 0.4±0.2c |
|  | MD-M | 0.1±0.0c | 1.1±1.6bc | 0.3±0.1c | 70±21c | 0.2±0.3b | 177±99b | 0.7±0.1c | 11±11b | 0.2±0.1c |
|  | MD-F | 1.2±0.1a | 8.2±0.7a | 6.0±1.2a | 229±2.7b | 5.5±0.1a | 728±119a | 5.4±1.1a | 19.4±7.1a | 1.3±0.1a |
|  | SARCO-F | 0.6±0.4b | 8.8±2.2a | 4.4±2.3b | 408±142a | 1.9±0.4b | 728±175a | 3.6±1.8b | 4.3±1.6bc | 0.9±0.2b |
| Khaothong | CM-M | 1.1±0.6b | 5.1±3.7a | 13±0.1b | 360±44a | 2.6±0.0ab | 283±107b | 12±0.5b | 9.5±0.9b | 1.8±0.3bc |
|  | CM-F | 1.0±0.4b | 3.4±0.6ab | 7.0±3.7bc | 387±70a | 4.2±2.8a | 448±178a | 5.6±3.3bc | 27±16a | 2.7±1.7ab |
|  | CR-M | 11±1.2a | 0.2±0.0b | 35±0.4a | 259±43b | 3.0±0.0ab | 151±0.2bc | 22±9.4a | 19±0.1a | 3.6±0.0a |
|  | CR-F | 0.9±0.5bc | 0.4±0.2b | 15±15b | 175±35c | 1.9±0.3bc | 241±39b | 8.1±6.9b | 6.1±4.4bc | 1.4±0.4c |
|  | MD-M | 0.1±0.0d | 0.7±0.2b | 0.3±0.1c | 22±1.2d | 0.4±0.0c | 27±4.9d | 0.1±0.0c | 0.5±0.1c | 0.2±0.1d |
|  | MD-F | 0.1±0.0d | 0.9±0.3b | 0.4±0.2c | 22±5.5d | 0.4±0.1c | 48±11cd | 0.2±0.1c | 0.6±0.3c | 0.2±0.1d |
|  | SARCO-F | 0.5±0.1c | 6.7±7.3a | 6.8±5.4bc | 235±111bc | 2.0±0.8bc | 506±125a | 7.4±7.1b | 5.2±3.0bc | 1.0±0.4cd |

CM = *Chrysomya megacephala*, CR = *Chrysomya rufifacies*, MD = *Musca domestica*, MS = *Musca sorbens*, ATH = *Atherigona* spp., SARCO = *Sarcophaga* spp., M = male, F = female. Values followed by the same letter are not significantly different (*p* > 0.05); lower-case letters show the difference of PTE concentrations among fly species (LSD: *p* < 0.05).
